# Brazilian Longitudinal Study of Adult Health (ELSA-Brasil): socio-occupational class as an effect modifier for the relationship between adiposity measures and self-rated health

**DOI:** 10.1186/s12889-019-7072-y

**Published:** 2019-06-11

**Authors:** Thaís Lopes de Oliveira, Rosane Harter Griep, Joanna Nery Guimarães, Luana Giatti, Dóra Chor, Maria de Jesus Mendes da Fonseca

**Affiliations:** 1 National School of Public Health, Oswaldo Cruz Foundation, Rua Leopoldo Bulhões, 1480, Manguinhos, Rio de Janeiro, RJ 21041-210 Brazil; 2 Laboratory of Health and Environment Education, Oswaldo Cruz Institute, Oswaldo Cruz Foundation, Avenida Brasil, 4365, Manguinhos, Rio de Janeiro, RJ 21040-360 Brazil; 3 Faculty of Medicine, Federal University of Minas Gerais, Belo Horizonte, 30310-100 Brazil

**Keywords:** Body mass index, Waist circumference, Self-rated health, Occupational social class, Social stratification, Effect modification

## Abstract

**Background:**

Little is known about the role of social class in the association between adiposity measures and self-rated health, and several studies have evaluated its influence as a confounder. The aim of the study is to investigate whether social class is an effect modifier in the association between adiposity measures and self-rated health in participants in the Brazilian Longitudinal Study of Adult Health (ELSA-Brasil).

**Method:**

Cross-sectional design, including 6453 men and 7686 women. Body mass index (kg/m^2^) and waist circumference (cms) were assessed. Self-rated health was categorized as good, fair and poor. Socio-occupational class was based on the participants’ occupation, education and per capita income. Multicovariate ordinal logistic model was used to evaluate the association between adiposity measures and self-rated health.

**Results:**

For women, the low and medium socio-occupational class effects were higher for those with waist circumference between 80 and 88 cm or overweight. For men, the low and medium socio-occupational class effects were higher for those with adequate waist circumference or normal body mass index.

**Conclusions:**

Social class is an effect modifier in the association between body mass index or waist circumference and self-rated health.

## Background

Self-rated health is considered one of the most relevant indicators for health research and It has been widely used as an indicator of health conditions in different populations [1, 2]. Several studies have demonstrated that self-rated health is a good predictor of morbidity and mortality, even after controlling for risk factors such as gender, race, marital status, and education [1, 3–5]. In addition to these associations, some factors are predominant in determining poor self-rated health. Among them are advanced age, female sex, low income, low educational level, unemployment, low social class, being married, having low social capital and being obese [2, 3, 6].

Obesity is a public health concern in Brazil; which prevalence has been increasing over the years [7]. Some research has shown that high values of body mass index (BMI) and waist circumference (WC) are associated with poor self-rated health [8–10]. Moreover, social stratification has been considered a potential explanation for differences in health [11].

Social class is a determinant of health [12], and being in a more distal level has an influence on body mass index and on the perception of health. Some studies have shown that the prevalence of inadequate BMI varies according to social class, with an excess of weight being more prevalent in lower social classes [13, 14]. Other studies have noted that people of lower social class rate their own health worse [6, 14, 15].

Due to the importance of social class in BMI and self-rated health, some authors treat the social class as a confounder variable in the relationship between adiposity measures and self-rated health [3, 16]. However, it is possible that this relationship is not homogeneous in all strata of social class [6, 11, 15], suggesting that this variable may act as an effect modifier.

An effect modification, also known as an interaction, can be described as “a situation in which two or more risk factors modify the effect of each other with regard to the occurrence or level of a given outcome” [17]. The investigation of the presence of interactions has important implications for public health, including important implications for prevention, for the planning of intervention and to identify the most vulnerable population groups [17, 18]. Nevertheless, the interaction is a phenomenon not often explored in the epidemiological literature [19].

Moreover, some studies have investigated interactions involving self-rated health as the main outcome [20–23], and, thus far, we have not found studies that evaluated the interaction between social class and adiposity measures in the association with self-rated health. In this way, the aim of this study was to investigate whether the social class is an effect modifier in the association between adiposity measures and self-rated health in participants from the Brazilian Longitudinal Study of Adult Health (ELSA-Brasil).

## Methods

### Study population

This study used baseline data (2008–2010) from ELSA-Brasil. The ELSA-Brasil is a multicentric cohort of civil servants (35–74 years) conducted at six study research centres in three regions of the country, including the Northeast, South, and Southeast. These centres are located at five federal universities and the Oswaldo Cruz Foundation [24]. The present study included 6453 men and 7686 women; participants who did not have information about weight, height or waist circumference measurements (*n* = 7) or did not answer the question about self-rated health (*n* = 4), socio-occupational class (*n* = 239) or other variables of interest to the study (*n* = 1) were excluded.

### Exposure

The exposures were body mass index (kg/m^2^) and waist circumference (cms). A stadiometer with a 0.1 cm scale was used to measure height. Weight was measured with the participant wearing a standardized uniform using a calibrated electronic scale with a capacity of 0 to 200 kg and divisions of 50 g. Waist circumference was measured using a measuring tape at the largest abdominal perimeter between the iliac crest and the last rib [25]. All measurements were performed using standardized techniques [26].

The following cut-off points adopted for BMI classification followed the recommendations of the World Health Organization [27]: ≤ 24.9 kg/m^2^ for underweight and normal weight, between 25 and 29.9 kg/m^2^ for overweight and ≥ 30 kg/m^2^ for obesity. The categories of underweight (≤ 18.5 kg/m^2^) and normal weight were grouped due to the small number of participants who were underweight (< 1%).

The WC cut-off points adopted for women were as follows: ≥ 80 cm and < 88 cm for increased risk of metabolic diseases and ≥ 88 cm for substantially increased risk. For males, the cut-off points were as follows: ≥ 94 cm and < 102 cm for increased risk of metabolic diseases and ≥ 102 cm for substantially increased risk [28].

### Outcome

Self-rated health [5] was obtained using the following question: “*In general, compared to people of your age, how do you consider your state of health?*”; the options were “*very good, good, fair, poor, or very poor*”. For the analyses, the answers were categorized as good self-rated health (very good and good), fair and poor self-rated health (poor and very poor).

### Effect modifier

**Fig. 1:**
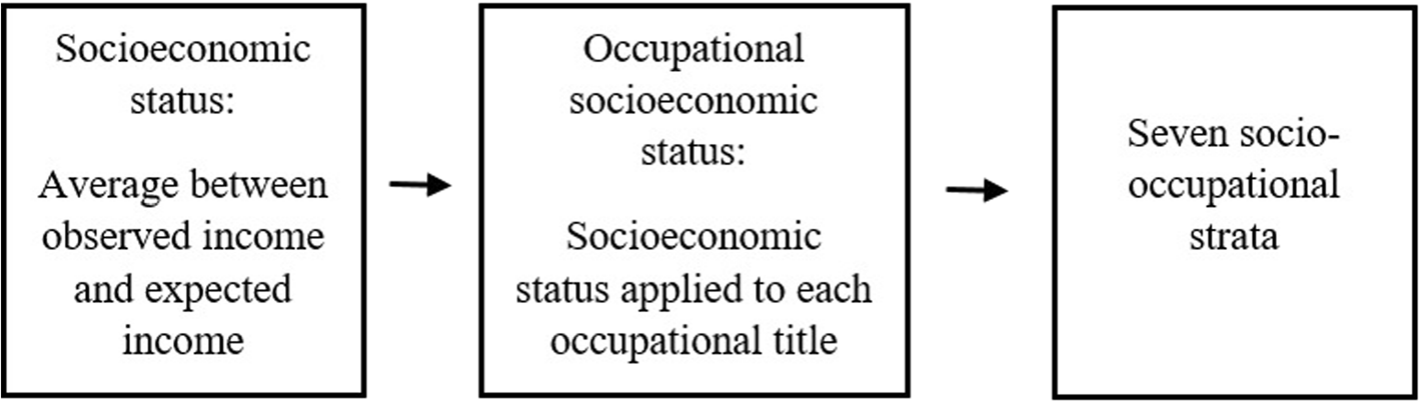
Diagram of socio-occupational class, ELSA-Brasil

Socio-occupational class was used as an indicator of social class. This variable was obtained from the participants’ socioeconomic status based on their described occupation, expected income (based on the level of education - average market value) and observed income [29]. Socioeconomic status was calculated as an average between observed income (economic component) and expected income (educational component). Subsequently, occupational socioeconomic status was calculated for each occupational title as the average score of the socioeconomic status of the individuals with different occupations (Fig. 1). Seven socio-occupational strata were defined based on the occupational socioeconomic status scores. These strata were divided into seven levels (upper-upper, upper-lower, upper-middle, middle-middle, middle-lower, lower-upper, and lower-lower) and ordered according to educational level and income associated with occupations. In this study, the strata were categorized as follows: high socio-occupational class (upper-upper and upper-lower), medium (upper-middle, middle-middle, middle-lower) and low (lower-upper and lower-lower).

### Covariables

**Fig. 2:**
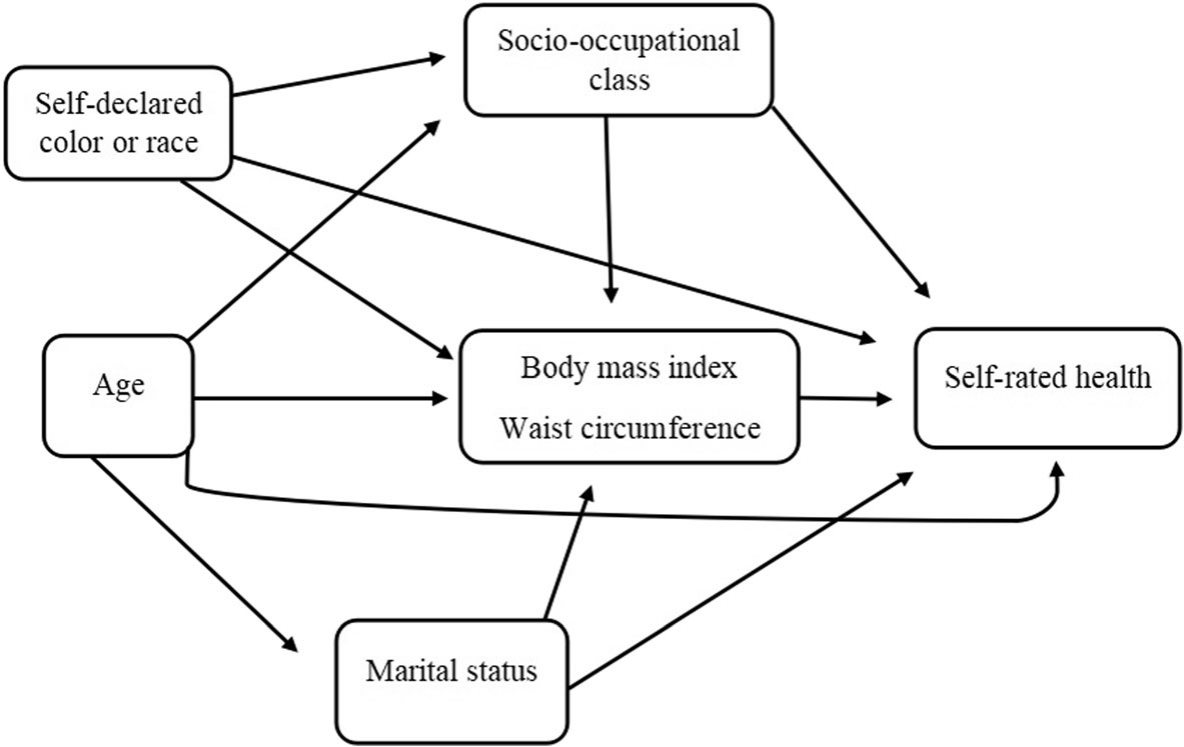
Directed acyclic graph of the relationship between adiposity measures and self-rated health - ELSA-Brasil

Directed acyclic graph (DAG) were constructed to represent the theoretical-operational model and to understand the involvement of covariables in the relationship between adiposity measures and self-rated health. The DAG was constructed using the Dagitty tool (available at: http://www.dagitty.net/dags.html). Additionally, the DAG was used to identify the minimum set of potential confounding variables. The covariates used in the DAG were age, self-declared colour or race, education, family income per capita, functional level, socio-occupational class, and marital status. The minimum set of potential confounding variables to explain the relationship between adiposity measures and self-rated health were socio-occupational class (low, medium and high), self-declared colour or race (white, brown and black), age and marital status (married/united, single, and separated/divorced/widowed/other) (Fig. 2).

### Statistical analyses

Means and standard deviations, and proportions were used to describe population characteristics regarding self-rated health. Through DAG, the selected variables were analysed using multicovariate models. We estimated the crude and adjusted association measurements (*Odds Ratios - OR*), obtained by single and multicovariate ordinal logistic model, respectively. This type of modelling tested the multiplicative interactions by the inclusion of an interaction term in the full adjusted model (BMI or WC*socio-occupational class). The effects of interactions were also illustrated.

All analyses were stratified by sex, and 95% confidence intervals were considered. The analyses were performed in the R software, version 3.5.1, library “MASS”, “epiDisplay”, “VGAM” and “effects”. The ELSA-Brasil study was approved by the research ethics committees of each of the institutions involved and all participants signed informed consent forms.

## Results

The mean age was similar for both sexes. The proportions showed that women with poor self-rated health were more likely to be married/united, brown self-declared colour, medium socio-occupational class, to have obesity and waist circumference above 88 cm. Men with poor self-rated health were more likely to be married/united, white self-declared colour, low socio-occupational class, overweight and waist circumference above 102 cm (Table 1).

**Table 1:** Characteristics of population regarding self-rated health - ELSA-Brasil baseline (2008–10)

	Self-rated health		
	Good	Fair	Poor
Female sex			
Age^a^	51.4 (8.8)	53.7 (8.9)	53.9 (8.6)
Self-declared colour^b^			
Black	1023 (16.6)	375 (27.8)	47 (27)
Brown	1617 (26.2)	468 (34.6)	64 (36.8)
White	3521 (57.1)	508 (37.6)	63 (36.2)
Marital status^b^			
Married/united	3328 (54)	671 (49.7)	81 (46.6)
Separated/divorced/widowed/other	1924 (31.2)	522 (38.6)	74 (42.5)
Single	909 (14.8)	158 (11.7)	19 (10.9)
Socio-occupational class^b^			
Low	1117 (18.1)	459 (34)	61 (35.1)
Medium	2955 (48)	669 (49.5)	87 (50)
High	2089 (33.9)	223 (16.5)	26 (14.9)
BMI^b^			
Obesity	1309 (21.2)	517 (38.3)	87 (50)
Overweight	2264 (36.7)	485 (35.9)	43 (24.7)
Normal weight	2588 (42)	349 (25.8)	44 (25.3)
Waist circumference^b^			
≥ 88 cm	2502 (40.6)	853 (63.1)	121 (69.5)
≥ 80 cm and < 88 cm	1722 [28]	291 (21.5)	32 (18.4)
Adequate	1937 (31.4)	207 (15.3)	21 (12.1)
Male sex			
Age^a^	51.6 (9.3)	53.9 (8.8)	54.7 (9.7)
Self-declared colour^b^			
Black	691 (13.3)	219 (18.8)	21 (21.2)
Brown	1547 (29.8)	420 (36.1)	35 (35.4)
White	2953 (56.9)	524 (45.1)	43 (43.4)
Marital status^b^			
Married/united	4249 (81.9)	970 (83.4)	76 (76.8)
Separated/divorced/widowed/other	653 (12.6)	146 (12.6)	21 (21.2)
Single	289 (5.6)	47 (4)	2 (2)
Socio-occupational class^b^			
Low	1312 (25.3)	494 (42.5)	46 (46.5)
Medium	1823 (35.1)	409 (35.2)	31 (31.3)
High	2056 (39.6)	260 (22.4)	22 (22.2)
BMI^b^			
Obesity	955 (18.4)	347 (29.8)	37 (37.4)
Overweight	2356 (45.4)	512 (44)	38 (38.4)
Normal weight	1880 (36.2)	304 (26.1)	24 (24.2)
Waist circumference^b^			
≥ 102 cm	1233 (23.8)	435 (37.4)	43 (43.4)
≥ 94 cm and < 102 cm	1385 (26.7)	305 (26.2)	23 (23.2)
Adequate	2573 (49.6)	423 (36.4)	33 (33.3)

^a^mean (standard deviation); ^b^ n (%)

Tables 2 and 3 show the crude and adjusted ORs (obtained by single and multicovariate models, respectively) treating the socio-occupational class as an effect modifier (adjusted OR). In crude analyses, individuals with overweight, obesity or higher waist circumference were more likely to report worse health than individuals with adequate BMI and WC.

**Table 2:** Multicovariate ordinal logistic model of the association between waist circumference and self-rated health - ELSA-Brasil (2008–10)

	Crude OR	Adjusted OR
Female sex		
Self-declared colour (white = 1)		
Brown	2.03 (1.78–2.31)	1.70 (1.48–1.95)
Black	2.53 (2.19–2.92)	1.89 (1.63–2.20)
Age (continuous variable)	1.02 (1.01–1.03)	1.02 (1.01–1.03)
Marital status (single = 1)		
Married/united	1.16 (0.97–1.39)	1.24 (1.03–1.50)
Separated/divorced/widowed/other	1.59 (1.33–1.92)	1.37 (1.13–1.67)
Waist circumference (adequate = 1)		
≥ 80 cm and < 88 cm	1.59 (1.33–1.91)	–
≥ 88 cm	3.31 (2.84–3.88)	–
Socio-occupational class (high = 1)		
Medium	2.15 (1.84–2.51)	–
Low	3.89 (3.29–4.60)	–
Interactions*		
Adequate WC* high socio-occupational class	–	1
≥ 80 cm WC* high socio-occupational class	–	0.95 (0.62–1.46)
≥ 88 cm WC* high socio-occupational class	–	2.51 (1.81–3.53)
Adequate WC* medium socio-occupational class	–	1.68 (1.19–2.39)
≥ 80 cm WC* medium socio-occupational class	–	2.62 (1.84–3.80)
≥ 88 cm WC* medium socio-occupational class	–	1.86 (1.52–2.29)
Adequate WC* low socio-occupational class	–	2.77 (1.86–4.16)
≥ 80 cm WC* low socio-occupational class	–	4.07 (2.79–6.04)
≥ 88 cm WC* low socio-occupational class	–	2.62 (2.10–3.28)
Male sex		
Self-declared colour (white = 1)		
Brown	1.53 (1.33–1.75)	1.30 (1.12–1.51)
Black	1.81 (1.52–2.15)	1.38 (1.14–1.65)
Age (continuous variable)	1.02 (1.02–1.04)	1.03 (1.02–1.04)
Marital status (single = 1)		
Married/united	1.46 (1.08–2.01)	1.14 (0.83–1.58)
Separated/divorced/widowed/other	1.53 (1.09–2.19)	1.31 (0.92–1.89)
Waist circumference (adequate = 1)		
≥ 94 cm and < 102 cm	1.34 (1.14–1.56)	–
≥ 102 cm	2.19 (1.89–2.53)	–
Socio-occupational class (high = 1)		
Medium	1.76 (1.49–2.07)	–
Low	2.99 (2.56–3.52)	–
Interactions*		
Adequate WC* high socio-occupational class	–	1
≥ 94 cm WC* high socio-occupational class	–	1.59 (1.13–2.23)
≥ 102 cm WC* high socio-occupational class	–	2.91 (2.15–3.96)
Adequate WC* medium socio-occupational class	–	2.20 (1.64–2.98)
≥ 94 cm WC* medium socio-occupational class	–	1.70 (1.22–2.37)
≥ 102 cm WC* medium socio-occupational class	–	1.96 (1.51–2.56)
Adequate WC* low socio-occupational class	–	3.93 (2.96–5.27)
≥ 94 cm WC* low socio-occupational class	–	3.15 (2.30–4.34)
≥ 102 cm WC* low socio-occupational class	–	2.22 (1.69–2.94)

*OR =* Odds Ratio, *WC =* Waist circumference; Ordinal logistic model was a proportional odds regression to model self-rated health (good, fair, poor). Crude and adjusted ORs were obtained by single and multicovariate models; Adjusted model: waist circumference + socio-occupational class + self-declared colour or race + age + marital status + waist circumference*socio-occupational class; * Effect of socio-occupational class status considering waist circumference

**Table 3:** Multicovariate ordinal logistic model of the association between body mass index and self-rated health- ELSA-Brasil (2008–10)

	Crude OR	Adjusted OR
Female sex		
Self-declared colour (white = 1)		
Brown	2.03 (1.78–2.31)	1.74 (1.51–1.99)
Black	2.53 (2.19–2.92)	1.88 (1.61–2.19)
Age (continuous variable)	1.02 (1.01–1.03)	1.03 (1.02–1.03)
Marital status (single = 1)		
Married/united	1.16 (0.97–1.39)	1.28 (1.06–1.54)
Separated/divorced/widowed/other	1.59 (1.33–1.92)	1.41 (1.16–1.71)
Body mass index (normal = 1)		
Overweigh	1.59 (1.33–1.91)	–
Obesity	3.31 (2.84–3.88)	–
Socio-occupational class (high = 1)		
Medium social class	2.15 (1.84–2.51)	–
Low social class	3.89 (3.29–4.60)	–
Interactions*		
Normal*high socio-occupational class	–	1
Overweigh*high socio-occupational class	–	1.01 (0.73–1.40)
Obesity*high socio-occupational class	–	2.51 (1.81–3.46)
Normal*medium socio-occupational class	–	1.75 (1.34–2.30)
Overweigh*medium socio-occupational class	–	2.36 (1.79–3.13)
Obesity*medium socio-occupational class	–	1.74 (1.32–2.29)
Normal*low socio-occupational class	–	2.68 (1.97–3.67)
Overweigh*low socio-occupational class	–	3.47 (2.59–4.69)
Obesity*low socio-occupational class	–	2.49 (1.86–3.36)
Male sex		
Self-declared colour (white = 1)		
Brown	1.53 (1.33–1.75)	1.26 (1.09–1.47)
Black	1.81 (1.52–2.15)	1.32 (1.09–1.58)
Age (continuous variable)	1.02 (1.02–1.04)	1.04 (1.03–1.04)
Marital status (single = 1)		
Married/united	1.46 (1.08–2.01)	1.13 (0.83–1.57)
Separated/divorced/widowed/other	1.53 (1.09–2.19)	1.29 (0.91–1.86)
Body mass index (normal = 1)		
Overweigh	1.34 (1.14–1.56)	–
Obesity	2.19 (1.89–2.53)	–
Socio-occupational class (high = 1)		
Medium social class	1.76 (1.49–2.07)	–
Low social class	2.99 (2.56–3.52)	–
Interactions*		
Normal*high socio-occupational class	–	1
Overweigh*high socio-occupational class	–	1.72 (1.25–2.41)
Obesity*high socio-occupational class	–	3.21 (2.26–4.59)
Normal*medium socio-occupational class	–	2.35 (1.68–3.32)
Overweigh*medium socio-occupational class	–	1.72 (1.33–2.22)
Obesity*medium socio-occupational class	–	1.94 (1.43–2.64)
Normal*low socio-occupational class	–	4.08 (2.95–5.73)
Overweigh*low socio-occupational class	–	2.89 (2.26–3.70)
Obesity*low socio-occupational class	–	2.15 (1.57–2.96)

*OR =* Odds Ratio, *BMI =* Body mass index; Ordinal logistic model was a proportional odds regression to model self-rated health (good, fair, poor). Crude and adjusted ORs were obtained by single and multicovariate models; Adjusted model: body mass index + socio-occupational class + self-declared colour or race + age + marital status + body mass index*socio-occupational class; * Effect of socio-occupational class status considering body mass index

In multicovariate model, the association measurements included the interaction term (BMI or WC*socio-occupational class). It is possible to notice, for both sexes, that interactions terms, for WC and BMI, had similar values and were significant (Tables 2 and 3, respectively). For women, the low and medium socio-occupational class effects were higher for those with WC between 80 and 88 cm (Table 2) or overweight (Table 3). For men, the low and medium socio-occupational class effects were higher for those with adequate WC (Table 3) or normal BMI (Table 2). For example, for women, the effect of the lower socio-occupational class, considering WC between 80 and 88 cm, in worsening self-rated health was 307% (OR = 4.07) higher than those of high socio-occupational class with adequate WC (Table 2). In addition, for men, the effect of the lower socio-occupational class, considering adequate WC, in worsening the self-rated health was 293% (OR = 3.93) higher than those of high socio-occupational class with adequate WC (Table 2).

**Fig. 3:**
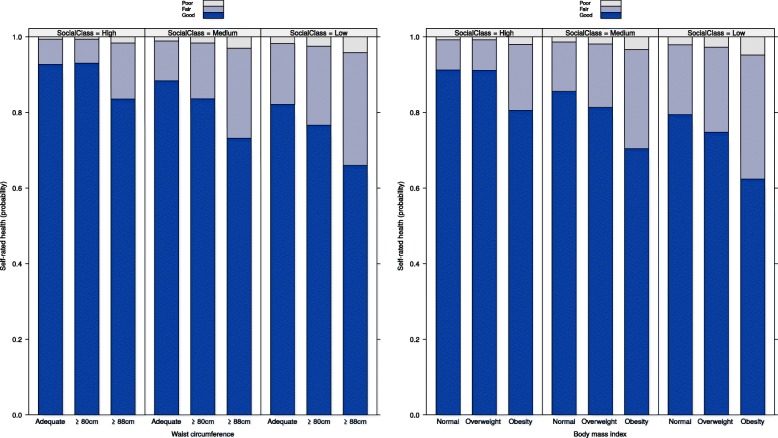
Effects of the BMI or WC and socio-occupational class interactions, female sex - ELSA-Brasil (2008–10). Note: WC = Waist circumference. BMI = Body mass index. Adjusted model: waist circumference or body mass index + socio-occupational class + self-declared colour or race + age + marital status + waist circumference or body mass index*socio-occupational class

**Fig. 4:**
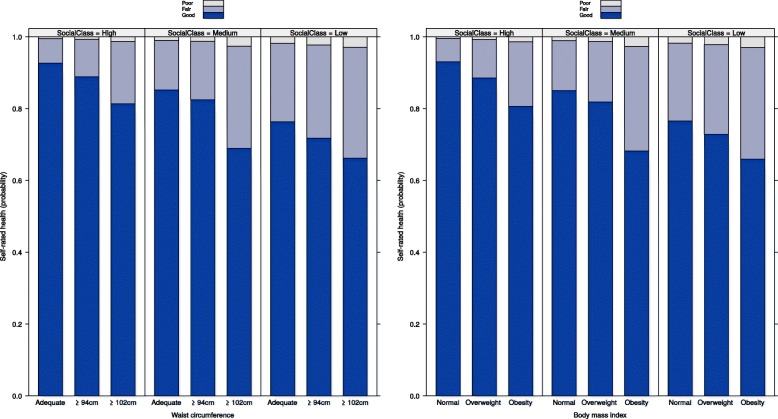
Effects of the BMI or WC and socio-occupational class interactions, male sex - ELSA-Brasil (2008–10). Note: WC = Waist circumference. BMI = Body mass index. Adjusted model: waist circumference or body mass index + socio-occupational class + self-declared colour or race + age + marital status + waist circumference or body mass index*socio-occupational class

In Figs. 3 and 4, the effects of the BMI and WC and socio-occupational class interactions, based on model 2, are shown. For both sexes, the probability of good self-rated health decrease with the increase of waist circumference, or body mass index, and with the decrease of socio-occupational class. Consequently, with the decrease in good self-rated health, the higher were the probabilities of fair or poor self-rated health.

## Discussion

In the analyses, we observed that some characteristics that influence worse self-rated health were similar for men and women, such as BMI and WC, socio-occupational class, self-declared colour or race, and marital status. Others studies conducted on a general population and with a worker population also found similar results [1, 3, 6, 14, 15, 30]. Moreover, in this study, we found that socio-occupational class behaved as an effect modifier of the association between BMI or WC and self-rated health.

Our results show the presence of interactions with significant effects for men and women. These results demonstrate that individuals exposed to a low socio-occupational class and inadequate BMI or WC had greater chances of worse self-rated health. There are few studies investigating the presence of an interaction between exposures and self-rated health. Knol et al. [19] conducted a systematic review to examine how interactions were studied and reported results from cohort and case-control studies, including studies from 2001 to 2007. The authors demonstrated the small number of articles with this purpose and reported that the most frequent exposures were treatments, medical conditions, and lifestyle factors, and the most common outcomes were cardiovascular disease, cancer and all causes of mortality.

To date, we have not found studies that evaluated an interaction between social class and adiposity measures in the association with self-rated health. However, we found two studies that studied interactions involving socioeconomic factors and other variables, with self-rated health as the main outcome. Both found important results with significant interactions.

Ahnquist and collaborators [20] found an interaction between economic capital and social capital when studying poor self-rated health in Sweden. Their results show that individuals exposed to socioeconomic difficulties and low social capital are more likely to self-rate their health as poor. These results corroborate ours, although they do not use the same variable to test the interactions; both the results (by this study and ours) demonstrate the importance of social class and socioeconomic conditions when studying self-rated health. Both studies found interactions with effects involving socioeconomic issues, in which people of a lower social class, and consequently with less education and income, self-evaluate their health worse.

In Germany, Trachte et al. [23] studied the presence of an interaction between physical activity and socioeconomic status (measured by education and income) in relation to self-rated health. The authors found, for females, a significant synergistic interaction between education and physical activity. Women with higher levels of education and physical activity rate their own health better. Once more, these studies demonstrate the importance of verifying the influence of issues involving socioeconomic conditions and life habits, such as physical activity, on self-rated health. In this way, in addition to health promotion and nutritional education policies, policies to reduce social inequality and promote social advancement may have an important role in reducing the effect of social class in the association between adiposity measures and self-rated health.

One of the limitations of the present study is the cross-sectional design, as the variables used in this study were measured at the same moment in time during the baseline interview. However, the variables selected to compose the proposed DAG are ancestral variables of self-rated health, reinforcing that the sectional design, in this case, was adequate to study interactions that influence fair/poor self-rated health [31].

Another limitation is the generalization of our findings to the non-worker population, as our results are from a cohort of civil servants. Nevertheless, if an interaction between BMI or WC and the socio-occupational class was present in the ELSA population, which includes individuals with employment and income, perhaps this effect is even greater in the general population, which is composed of unemployed individuals with lower levels of education and income.

The large number of participants in the ELSA-Brasil study can be cited as an advantage of this study, given the need to have large sample sizes for the study of interactions [32, 33]. Kamangar [33] showed that a study with the aim of detecting interactions would require more than twice as many participants when compared to a study without this objective. Therefore, ELSA-Brasil had an adequate sample that allowed for the identification of variables that behave as effect modifiers.

Another advantage of this study was the use of two anthropometric measurements, BMI and WC. The association of different anthropometric methods assists in the nutritional diagnosis and can reduce the classification error associated with the use of just one anthropometric measurement [27]. Additionally, all equipment used was periodically checked; the scales were calibrated, the measuring tapes evaluated, and all interviewers were periodically recertified [34].

## Conclusions

Therefore, the results show that the combined effects of social class and BMI or WC are more important than the independent effects of these factors on self-rated health. Our findings call attention to a more vulnerable population subgroup in relation to worse self-rated health, that is, those with overweight/obesity and low socioeconomic level. Nevertheless, these results can help in the formulation of public policies that involve adiposity measures, social class, social inequality, and other important issues when studying self-rated health.

## Data Availability

The datasets used and analysed during the current study are available from the corresponding author on reasonable request.

## Abbreviations

BMI: Body mass index
DAG: Directed acyclic graph
ELSA-Brasil: Brazilian Longitudinal Study of Adult Health
OR: Odds Ratios
WC: Waist circumference
